# Prevalence of musculoskeletal disorders and associated risk factors in canadian university students

**DOI:** 10.1186/s12891-023-06630-4

**Published:** 2023-06-19

**Authors:** Dorsa Nouri Parto, Arnold YL Wong, Luciana Macedo

**Affiliations:** 1 Michael G. DeGroote School of Medicine, McMaster University, Hamilton, Canada; 2 Department of Rehabilitation Sciences, The Hong Kong Polytechnic University, Hong Kong SAR, China; 3 School of Rehabilitation Sciences, McMaster University, 1400 Main St West IAHS 441, Hamilton, ON Canada

**Keywords:** Musculoskeletal disorders, University students, Psychological stress, Young adults

## Abstract

**Background:**

Musculoskeletal disorders (MSKDs) present a significant burden to health care systems worldwide. Evidence suggests that university students may have unique risk factors for developing MSKDs; however, research on the corresponding prevalence and risk factors of MSKDs in Canadian students is limited.

**Methods:**

Using a multi-year cross-sectional survey, we aimed to understand the prevalence and risk factors of MSKDs in students at McMaster University. A survey on the prevalence of MSKD as well as potential risk factors was conducted online in the years 2018–2022. Our outcomes were the prevalence of MSKDs over the last 7 days and the last 12 months, as well as presence of lower body, upper body, and spine MSKDs. We investigated risk factors using negative binomial regression analysis, including a sex-stratified analysis.

**Results:**

There were a total of 289 respondents in 2018 with a decrease in the number of participants in the subsequent years (n_2019_ = 173, n_2020_ = 131, n_2021_ = 76). Participants reported a median of 2–3 pain sites in the last year and 1–2 pain sites in the last week in all four years. The most prevalent sources of self-reported pain were the lower back and neck. Depending on the year and outcome studied, 59–67% of participants reported neck/lower back pain in the last year, and 43–49% reported it in the last week. Although risk factors were different depending on the year and sex, overall, poorer mental health, being in health care studies, regular sports participation (males only), older age, and less hours of sleep were significantly associated with higher prevalence of MSKDs.

**Conclusions:**

This study identified that MSKDs are a prevalent source of pain in university students. While some risk factors, such as mental health, are known to play a role in developing MSKDs, sports activity and academic pressure are risk factors that are unique to students. Our study also suggests that there may be differences in risk factors between sexes.

**Supplementary Information:**

The online version contains supplementary material available at 10.1186/s12891-023-06630-4.

## Background

One in five Canadians experience chronic pain, which costs the Canadian economy up to $60 billion a year in health care costs and lost wages and taxes, more than cardiovascular disease, cancer, and diabetes combined. Canada has a high prevalence of musculoskeletal disorders (MSKDs), with point prevalence estimates as high as 27.8% in 2017, with the highest prevalence in the back and knee joints [1]. MSKDs may present as acute (< 4 weeks), subacute (4 to 12 weeks), or chronic pain (> 12 weeks) [2]. One main risk factor for transition to chronic pain is a previous episode of pain [3]. Risk factors for MSKDs can differ depending on the individual’s age and occupation [4].

Previous research suggests that being a university student can present with unique risk factors for developing MSKDs. For example, increased screen time and prolonged sitting, compounded with high levels of stress, can act as risk factors for the development of MSKDs in university students [4]. Other suggested risk factors for MSKDs include the type of electronic device used for studying and the position in which it is used [5, 6]. In addition, there is evidence to suggest that the field of study can also influence the likelihood of developing MSKDs, as university students in the health care studies have a higher risk of MSKDs compared to those in non-health care studies [7–9]. For example, studies have shown a higher prevalence of temporomandibular disorders and associated headache pain in females in the health and sciences field [10]. Furthermore, exercise is often reported as a protective factor against MSKDs; however, previous research in undergraduate students suggests that involvement in competitive sports can increase the likelihood of knee pain [11]. There is also a significant body of literature suggesting an association between mental health and MSKDs [12]. With a growing prevalence of mental health problems and stress in university students, this transition into adulthood is a vulnerable time for students to develop MSKDs [12, 13].

Despite the potential consequences of MSKDs and their high prevalence in the Canadian population, risk factors for developing MSKDS in university students are an under-explored area of study. The goal of our study was to better understand MSKD risk factors in a Canadian university setting, as well as the distribution of MSKDs in different body regions. Based on previous studies, we hypothesized that the neck and the lower back region will be the most common sites of MSKDs. We also hypothesized that certain factors were associated with the presence of MSKDs, including poor mental health, older age, being a healthcare student, increased screen time, and high study pressure.

## Methods

### Study design

This was a multi-year cross-sectional study survey of McMaster University students in the years 2018, 2019, 2020, and 2021, originally designed with the aim of conducting a longitudinal study. University students were invited to complete a survey in 2018 and respondents were asked to complete the same survey in the three subsequent years. However, given that many respondents did not provide an email address to allow matching between survey years, we were unable to cross-reference participants and perform a longitudinal study. As such, we chose to perform a cross-sectional analysis.

### Setting

Following ethics approval, faculties and programs from McMaster University were identified through the university website. We sent invitation emails to 44 faculties, departments or programs within the university. A total of 24 faculties, departments or programs (55% of those reached) agreed to send our study advertisement to their student mailing lists. Three rounds (weekly) of invitation/advertisement emails were sent to students in September 2018. Survey invitations were sent out again in September 2019, 2020, and 2021 to only those that responded to the initial survey and shared their email address. Participation in this survey was voluntary and respondents entered a prize draw as an incentive. There were approximately 31,000 students enrolled at McMaster University, although it is unclear how many students were reached through our distribution system.

### Inclusion and exclusion criteria

All McMaster University course-based students (undergraduate or graduate) aged 17 years or older were eligible for inclusion regardless of the year of study. Students were excluded from this survey if they were studying research-based graduate programs or were unable to provide program of study.

### Survey

Surveys were completed using a commercially available private survey platform named “Typeform PRO” (https://www.typeform.com/). The questionnaire comprised a maximum of 88 questions. The questionnaire was developed by a panel of clinicians and scientists with over 18 years of experience in clinical practice or conducting musculoskeletal research. The questionnaire has been used in similar epidemiological research for university students [11]. Specifically, it consisted of three sections: the first section included a modified Nordic Musculoskeletal Questionnaire to assess musculoskeletal symptoms in nine body parts (i.e., neck, shoulder, elbow, wrist/fingers, upper back, lower back, hip/thigh, knee and ankle) at the present moment, and in the last 7 days and the last 12 months [14]. The second section solicited information related to demographics, potential risk factors for musculoskeletal disorders such as smoking or drinking history, types of work surface and school bags, types of sport participation, physical activity levels, daily duration of cell phone usage or computer usage, and the total number of lecture hours. The third section included the Depression Anxiety Stress Scales-14. Using skip logic, respondents could skip irrelevant questions based on their responses to previous questions. The average completion time was approximately 14 min.

### Outcomes

Outcome measures included the total number of pain sites in each participant over the last week and the last 12 months in each year surveyed, as well as presence of MSKD pain at the lower body, upper body, and spine. Participants reported on the presence of MSKDs in each body part (head, neck, upper back, lower back, shoulders, elbows, wrist/hand, pelvis/groin, hips/tights, knees, lower legs, or ankles/feet/toes). This outcome was reported as a yes or no for each participant for each time point. The total number of MSKD pain sites at a given time point was calculated by summing all the sites with pain. The total number of MSKD sites ranged from 0 to 11.

In addition to the estimation of the total number of pain sites, MSKD pain was grouped into three categories: (1) *upper body*, consisting of shoulder, elbow, wrist/fingers and neck; (2) *lower body*, including hip/thigh, knee, and ankles; and (3) *spine*, which included both upper and lower back injuries. A yes or a no were assigned to each of the three sites depending on the presence or absence of an injury at each time point.

### Predictors

We identified potential predictors that had been reported in the literature to be associated with MSK pain such as depression and anxiety [15], as well as potential university student-specific factors.

#### Depression and anxiety

we used questions from Depression Anxiety Stress Scale-14 (DASS-14) to assess depression and anxiety [16]. Responders were asked to choose the degree to which criteria such as mouth dryness, non-positive feelings, and difficulty breathing applied to them. The degrees of applicability ranged from 1 to 4, with 4 being the most applicable. A total score was calculated by summing all 14 questions, with higher scores reflecting poorer mental health status.

#### Overall pressure score

Participants rated the level of study pressure, family pressure, and peer pressure that they experienced from 0 to 10, with 10 being most severe. The pressure ratings from these three categories were summed to create a pressure score predictor, ranging from 0 to 30.

#### Program of study

students were asked to choose their program of study from the list of options or provide another program of study if a corresponding one was not listed. Previous research has suggested that healthcare students are more likely to have MSKDs [5], In the current study, the academic programs were classified as healthcare (including medicine, speech-language pathology, physiotherapy, Bachelor of Health Sciences, midwifery, and nursing), or non-healthcare (engineering, science, arts, social work, and arts and science). It was considered as a predictor variable.

#### Student-related factors

Student related predictor variables included *hours of part-time work* per week, *hours of computer and cell phone use* per day, type of computer used (i.e., laptop), work surface (i.e., desk), and type of school bag (i.e., backpack).

#### Average hours slept per night

Respondents were asked to estimate their average hours of sleep per night in the last week. Sleep problems have been routinely associated with higher MSK pain, and poor sleep has been suggested to contribute to fibromyalgia [17].

#### Physical activity

Responders were also asked if they engage in regular sports activity, and were asked to respond yes or no.

#### Smoking

Responders were asked whether they smoked, with four possible responses (“Yes”, “Yes but at rare occasions”, “No but I have smoked before”, and “No, I do not smoke”).

### Statistical analysis

#### Prevalence of MSKDs

The 7-day and 12-month prevalence was reported as the number of participants who indicated pain per body site over the last 7 days and the past 12 months at a given time point.

#### Total number of pain sites

Two predictive models (pain over the last week, pain over the past year) were constructed for each year to assess factors associated with the total number of pain sites. A negative binomial mixed effect model was conducted due to the count nature and over dispersion of the dependent variables. All analyses were conducted for the overall population and then stratified by sex to assess for differences in musculoskeletal injuries between men and women [18].

The distributions of all predictors were assessed to ensure sufficient variability for analysis. Previous studies in university students have suggested that smoking is a moderate risk factor for MSK injuries [19]; however, we did not include smoking in our model due to little variability in responses. More than 90% of students indicated that they were non-smokers. Similarly, type of school bag (backpack), computer used (laptop), and work surface (desk) corresponded to more than 90% of the responses and could not be used as predictors. Age and academic year of study were highly correlated in 2018 (*r*(285) = 3.85, *p <* 0.0001); therefore, we only included academic year of study in 2018 due to its better fit to the model. In 2019, the two predictors were not correlated (*r*(168) = 1.53, *p* = 0.12) and were both included in the model.

We used a backward elimination method to build the models; variables were sequentially removed based on the probability score to reach the model with the lowest Akaike information criterion (AIC) score. We chose to use the AIC in place of the Bayesian information criterion (BIC), because BIC tends to choose models that are too simple for finite samples [20]. An AIC score difference of 2 or higher was considered significant enough to justify the removal of a variable for a simpler model. All statistical analyses were performed in R 4.0.2 using package *MASS.*

#### Presence of lower body, upper body or spine pain

We built models to assess *yearly* risk factors for spine, upper body, and lower body injuries separately. Since the dependent variable was the presence or absence of pain in a given body region, a binomial logarithmic regression analysis was performed. The risk factors were assessed as a combined dataset initially, and subsequently stratified for men and women separately. Variables were removed in the order of highest P-value to create models with risk factors within 0.1 probability. This analysis was only done for the year 2018 due to the small sample size in other years.

## Results

There were 298 respondents to our survey in 2018, but the numbers gradually decreased with each sampling year (n_2019_ = 173, n_2020_ = 131, and n_2021_ = 76). In 2018 and 2019, 20 and 10 respondents were excluded respectively from the analysis due to either not responding to majority of the questions or not meeting the inclusion criteria (e.g., not providing a field of study or being enrolled in a research-based program). Approximately 70% of respondents each year were females. The average age of participants in the first responding year was 21.9 years old (SD 3.80) ranging from 17 to 47 years. Characteristics of the participants are shown in Table 1. Sex-stratified characteristics can be found in Table 1 A [see additional file 1].

**Table 1:** Description of the predictor variables

Sociodemographic data	Year 2018 (n = 298)	Year 2019 (n = 173)	Year 2020 (n = 131)	Year 2021 (n = 76)	
Mean age (in years)	21.9 ± 3.8	22.9 ± 3.9	23.8 ± 3.4	24.6 ± 3.5	
Sex (Female)	209 (70.1%)	120 (69.4%)	88 (67.1%)	54 (71.1%)	
Hours of part-time work per week	5.2 ± 9.3	2.3 ± 9.3	3.8 ± 6.6	4.4 ± 8.6	
Year of study (0, 1, 2, 3, 4)*					
0	65 (21.8%)	6 (3.5%)	1 (0.76%)	2 (2.6%)	
1	73 (24.5%)	48 (27.7%)	6 (4.6%)	5 (6.6%)	
2	49 (16.4%)	51 (28.5%)	44 (33.6%)	3 (3.9%)	
3	62 (20.8%)	37 (21.4%)	36 (27.5%)	17 (22.4%)	
4	33 (11.1%)	18 (10.4%)	24 (18.3%)	23 (30.3%)	
> 5	12 (4.0%)	11 (6.3%)	19 (14.5%)	21 (27.6%)	
Type of study program					
Healthcare	142 (47.7%)	91 (52.6%)	72 (55.0%)	42 (55.3%)	
Non-healthcare	153 (51.3%)	80 (46.2%)	59 (45.0%)	34 (44.7%)	
Habits					
Regular exercise (Yes)	156 (52.3%)	86 (49.7%)	55 (42.0%)	30 (39.5%)	
Average hours of sleep over the last week	6.8 ± 1.5	6.6 ± 1.2	7.1 ± 1.4	6.7 ± 1.0	
h of computer usage per day	5.8 ± 3.2	5.6 ± 2.8	6.0 ± 3.1	5.8 ± 2.9	
h of cellphone usage per day	3.2 ± 2.6	3.2 ± 2.2	3.8 ± 2.7	3.4 ± 1.9	
Psychological factors					
Depression, Anxiety Stress Scale DASS-14 (14–30)	24.5 ± 7.8	22.1 ± 6.9	22.7 ± 7.4	20.4 ± 6.7	
Total pressure score from family, peer, and studies (1–30)	15.3 ± 6.0	14.5 ± 6.2	14.8 ± 5.9	13.4 ± 6.0	

* Year of study refers to year of study in the last academic year. 0 was used for students that were below first year of study in the last year

In the past 12 months, respondents reported a median of 3 pain sites in years 2018 (IQR = 3), 2019 (IQR = 4), and 2021 (IQR = 3), while a median of 2 (IQR = 3) pain sites in 2020 (Table 2). Sex-stratified prevalence of pain sites can be found in Table 3 A [Additional file 1].

With regards to MSKDs in the past week, respondents reported a median of 2 pain sites in year 2018 (IQR = 1), 1 pain site in 2019 and 2020 (IQR = 3), and a median of 2 pain sites in 2021 (IQR = 2).

The prevalence of pain sites in head, neck, upper back, lower back, shoulders, elbows, wrist/hand, pelvis/groin, hips/tights, knees, lower legs, ankles/feet/toes have been summarized on Table 2 A [Additional file 1]. The lower back was the most prevalent pain site in all four years; 40.5–55.2% of all respondents had at least one episode of lower back pain in the last 12 months.

In terms of distribution by body region, lower body injuries were the least commonly-reported yearly injuries in all four years, as compared to the spine and upper body, with the year 2020 having the least prevalence of yearly lower body injuries (46.6% of respondents) (Table 2). In terms of weekly prevalence of injuries, upper body injuries were the most prevalent in 2018 and 2019 with more than 70% of respondents reporting an MSKD in the past week, but there was a downward trend in its prevalence in 2020 and 2021 to 52.6% and 38.2%, respectively. Weekly spine and lower body injuries also displayed a downward trend in prevalence in 2020 and 2021.

**Table 2:** Summary of number of pain sites in all four years

	Year 2018 (n = 298)		Year 2019 (n = 173)		Year 2020 (n = 131)		Year 2021 (n = 76)	
	Median	(25th – 75th percentile)	Median	(25th – 75th percentile)	Median	(25th – 75th percentile)	Median	(25th – 75th percentile)
MSKD counts (yearly)	3	2–5	3	1–5	2	1–4	3	1–4
MSKD counts (weekly)	2	1–3	1	0–3	1	0–3	2	1–2
Yearly prevalence of pain by body region								
Spine (Yes) n(%)	193 (64.8%)		107 (61.8%)		82 (62.6%)		52 (68.4%)	
Lower Body (Yes) n(%)	178 (59.7%)		93 (53.8%)		61 (46.6%)		40 (52.6%)	
Upper Body (Yes) n(%)	191 (64.1%)		110 (63.6%)		81 (61.8%)		50 (65.8%)	
Weekly prevalence of injury by body region								
Spine (Yes) n(%)	175 (58.7%)		90 (52.0%)		52 (39.7%)		37 (48.7%)	
Lower Body (Yes) n(%)	194 (65.1%)		90 (52.0%)		38 (29.0%)		32 (42.1%)	
Upper Body (Yes) n(%)	221 (74.2%)		140 (80.9%)		69 (52.6%)		29 (38.2%)	

### Regression analysis

Initially, we planned to assess risk factors for pain sites in all four years; however, as years 2020 and 2021 had very low samples sizes, risk factors were only assessed for years 2018 and 2019.

### Yearly MSKD counts − 2018

Yearly MSKD counts represented the sum of total pain sites in an individual in the last year. The results of regression model for yearly pain sites for 2018 are listed in Table 3. In the year 2018, higher DASS-14 score (IR = 1.02, CI: 1.01–1.03), being a health care student (IR = 1.21, CI: 1.02–1.44), and higher hours of cell phone browsing (IR = 1.02, CI: 1.01–1.03) were significantly associated with higher counts of yearly pain sites. The R^2^_Nagelkerke_ value was 0.10 for the overall model. *When stratified by sex*, females and males had some differences in significant predictor variables. In females, higher DASS-14 score (IR = 1.02, CI: 1.01–1.03, R^2^_Nagelkerke_ = 0.11) was associated with higher yearly MSKDs, while being in a higher academic year of study was associated with decrease in MSKDs (IR = 0.93, CI: 0.88–1.00). For males in 2018, having a regular sports activity was associated with 55% increase in the number of yearly MSKDs (IR = 1.55, CI: 1.06–2.28, R^2^_Nagelkerke_ = 0.16).

**Table 3:** Risk factors for yearly count of pain sites in 2018

Year 2018						
	Overall (n = 278, R^2^_Nagelkerke_ = 0.10)		Female (n = 201, R^2^_Nagelkerke_ = 0.11)		male (n = 77, R^2^_Nagelkerke_ = 0.16)	
**Predictors**	*Incidence Ratio* *(Confidence Interval)*	*P-value*	*Incidence Ratio* *(Confidence Interval)*	*Pr(>|z|)*	*Incidence Ratio* *(Confidence Interval)*	*Pr(>|z|)*
Mental health (DASS-14)	**1.02** **(1.01–1.03)**	< 0.001*	**1.02** **(1.01–1.03)**	0.001*	-	*-*
Study pressure	0.99 (0.98–1.00)	*0.161*	0.99 (0.98–1.01)	*0.498*	0.98 (0.95–1.01)	*0.137*
Health care student (Yes)	**1.21** **(1.02–1.44)**	0.03*	1.21 (0.99–1.48)	*0.061*	- -	*-*
Hours of part-time work per week	1.01 (1.00–1.01)	*0.197*	1 (0.99–1.01)	*0.661*	1.02 (1.00–1.05)	*0.058*
Last academic year of study (1–5+)	0.95 (0.90–1.00)	*0.07*	**0.93** **(0.88–1.00)**	0.037*	-	*-*
Last week’s average sleep	1.0 (0.96–1.03)	*0.955*	-	*-*	-	*-*
Hours of cell phone browsing	**1.02** **(1.01–1.03)**	< 0.001*	0.99 (0.95–1.02)	*0.473*	-	*-*
Hours of computer use	-	*-*	-	*-*	-	*-*
Regular sport activity (Yes)	1.1 (0.93–1.30)	*0.287*	-	*-*	**1.55** **(1.06–2.28)**	0.026*

* P < 0.05

### Weekly MSKD counts − 2018

Factors associated with weekly MSKD counts are listed in Table 4. Overall, the DASS-14 score was the only variable that had a significant association with weekly pain sites in the year 2018 (IR = 1.03, CI: 1.01–1.04, R^2^_Nagelkerke_ = 0.10). *When the analysis was stratified by sex, an* DASS-14 score was associated with an increase in MSKDs in females (IR = 1.03, CI: 1.01–1.04, R^2^_Nagelkerke_ = 0.14). In men, having a regular sports activity was associated with an increased count of MSKDs by 72% (IR = 1.72, CI: 1.07–2.81, R^2^_Nagelkerke_ = 0.09).

**Table 4:** Risk factors for weekly count of pain sites − 2018

Year 2018						
	Overall (n = 278, R^2^_Nagelkerke_ = 0.10)		Female (n = 201, R^2^_Nagelkerke_ = 0.14)		Male (n = 78, R^2^_Nagelkerke_ = 0.09)	
**Predictors**	*Incidence Ratio* *(Confidence Interval)*	*P-value*	*Incidence Ratio* *(Confidence Interval)*	*Pr(>|z|)*	*Incidence Ratio* *(Confidence Interval)*	*Pr(>|z|)*
Mental health (DASS-14)	**1.03** **(1.01–1.04)**	< 0.001*	**1.03** **(1.01–1.04)**	< 0.001**	-	*-*
Study pressure	1 (0.98–1.02)	*0.97*	1.00 (0.98–1.02)	0.673	-	*-*
Health care student (Yes)	1.01 (1.00–1.02)	*0.073*	-	-	-	*-*
Hours of part-time work per week	0.96 (0.89–1.03)	*0.221*	1.01 (1.00–1.02)	0.120	1.01 (0.98–1.04)	*0.401*
Last academic year of study (1–5+)	1.03 (1.01–1.04)	*0.97*	0.93 (0.86–1.01)	0.081	-	*-*
Last week’s average sleep	-	*-*	-	-	-	*-*
Hours of cell phone browsing	1 (0.96–1.04)	*0.903*	0.98 (0.94–1.03)	0.476	-	*-*
Hours of computer use	-	*-*	-	-	-	*-*
Regular sport activity (Yes)	1.11 (0.90–1.36)	*0.35*	-	-	1.72* **(1.07–2.81)**	0.029*

* P < 0.05

### Yearly MSKD counts – 2019

The results of regression analysis for yearly MSKDs in 2019 are similar to the results in 2018 (see Table 5) In 2019, higher DASS-14 score (IR = 1.03, CI: 1.01–1.04) and higher academic year of study (IR = 1.02, CI: 1.003–1.04) were significantly associated with an increased count of yearly pain sites (R^2^_Nagelkerke_ = 0.06). *When stratified by sex* the only significant predictor for female yearly MSKDs was the DASS-14 score. An increase in DASS-14 score was significantly associated with higher number of pain sites in both females (IR = 1.02, CI: 1.004–1.05, R^2^_Nagelkerke_ = 0.08) and males (IR = 1.04, CI: 1.04–1.09, R^2^_Nagelkerke_ = 0.32). In males, in addition to the DASS-14 score and an increase in age by a year was associated with approximately 16% increase in MSKDs (OR = 1.16, CI: 1.03–1.32). Interestingly, an increase in study pressure was associated with a significant decrease in number of pain sites (IR = 0.96, CI: 0.93–0.996).

**Table 5:** Risk factors for yearly counts of pain sites in 2019

Year 2019						
	Overall (n = 163, R^2^_Nagelkerke_ = 0.06)		Female (n = 112, R^2^_Nagelkerke_ = 0.08)		Male (n = 45, R^2^_Nagelkerke_ = 0.32)	
**Predictors**	*Incidence Ratio* *(Confidence Interval)*	*P-value*	*Incidence Ratio* *(Confidence Interval)*	*Pr(>|z|)*	*Incidence Ratio* *(Confidence Interval)*	*Pr(>|z|)*
Mental health (DASS-14)	**1.02** **(1.01–1.04)**	0.043*	**1.02** **(1.004–1.05)**	0.039*	**1.04** **(1.004–1.09)**	0.048*
Study pressure	1 (0.97–1.02)	*0.897*	1.01 (0.98–1.04)	0.454	**0.96** **(0.93–0.996)**	0.035*
Health care student (Yes)	1.17 (0.85–1.60)	*0.33*	1.2 (0.87–1.66)	0.26	-	*-*
Hours of part-time work per week	1.02 (0.91–1.13)	*0.743*	-	-	0.99 (0.94–1.03)	*0.634*
Last academic year of study (1–5+)	**1.02** **(1.003–1.04)**	0.043*	0.96 (0.84–1.09)	0.503	1.12 (0.95–1.33)	*0.178*
Last week’s average sleep	-	*-*	-	-	-	*-*
Hours of cell phone browsing	1.01 (0.95–1.08)	*0.752*	1 (0.94 –1.08)	0.939	-	*-*
Hours of computer use	0.99 (0.94–1.04)	*0.608*	0.99 (0.93–1.05)	0.737	-	*-*
Regular sport activity (Yes)	-	***-***	-	-	-	*-*
Age	1.01 (0.97–1.05)	*0.643*	-	-	**1.16** **(1.03–1.32)**	0.016*

* P < 0.05

### Weekly MSKD counts – 2019

Weekly MSKDs in 2019 are summarized in Table 6. Overall, DASS-14 score was the only variable with a significant correlation with weekly pain sites in 2019 (IR = 1.02, CI: 1.004–1.05, R^2^_Nagelkerke_ = 0.05). *When stratified by sex*, for females, DASS-14 was the only predictor with a significant relationship to MSKDs. An increase in DASS-14 score was associated with 3% increase in MSKDs (IR = 1.03, CI: 1.004–1.06, R^2^_Nagelkerke_ = 0.12). This relationship was also seen in males (IR = 1.05, CI: 1.002–1.11, R^2^_Nagelkerke_ = 0.32). In addition, higher study pressure was associated with a smaller number of weekly pain sites (IR = 0.95, CI: 0.92–0.99). Lastly, an increase in age was associated with 21% increase in the number of pain sites (OR = 1.21, CI: 1.05–1.40).

**Table 6:** Risk factors for weekly counts of pain sites in 2019

Year 2019						
	Overall (n = 163, R^2^_Nagelkerke_ = 0.05)		Female (n = 112, R^2^_Nagelkerke_ = 0.12)		Male (n = 45, R^2^_Nagelkerke_ = 0.32)	
**Predictors**	*Incidence Ratio* *(Confidence Interval)*	*Pr(>|z|)*	*Incidence Ratio* *(Confidence Interval)*	*Pr(>|z|)*	*Incidence Ratio* *(Confidence Interval)*	*Pr(>|z|)*
Mental health (DASS-14)	**1.02** **(1.004–1.05)**	0.049*	**1.03** **(1.004–1.06)**	0.049*	**1.05** **(1.002–1.11)**	0.029*
Study pressure	1.01 (0.99–1.04)	*0.349*	1.03 (1.00–1.07)	0.059	**0.95** **(0.92–0.99)**	0.015*
Health care student (Yes)	1.22 (0.83–1.82)	*0.299*	1.17 (0.76–1.64)	0.407	-	*-*
Hours of part-time work per week	1 (0.97–1.03)	*0.981*	-	-	1.02 (0.96–1.07)	*0.507*
Last academic year of study (1–5+)	-		1.01 (0.87–1.16)	0.942	1.06 (0.87– 1.28)	*0.570*
Last week’s average sleep			-	-	-	*-*
Hours of cell phone browsing	1 (0.92–1.07)	*0.897*	0.98 (0.91–1.07)	0.707	-	*-*
Hours of computer use	0.99 (0.94–1.05)	*0.819*	0.99 (0.93–1.07)	0.860	-	*-*
Regular sport activity (Yes)			-	-	-	*-*
Age	0.99 (0.94–1.04)	*0.757*	-	-	**1.21** **(1.05–1.40)**	0.010*

* P < 0.05

### Predictors of yearly spine, upper body, and lower body injuries − 2018

Yearly prevalence of upper and lower body and spine MSKDs logistic regression are summarized in Table 7 (Tables 7 and 8 A provide more details of the regression).

1. Upper body.

Overall, higher DASS-14 scores (OR = 1.05, CI: 1.01–1.09, R^2^_Tjur_ = 0.09), being a health care student (OR = 2.51, CI: 1.48–4.35), lower academic year of study (OR = 0.77, CI: 0.64–4.35) and having a regular sports activity (OR = 1.84, CI: 1.10–3.11) were associated with higher likelihood of upper body injuries. R^2^_Tjur_ value was 0.09. *When stratified by sex*, in females, an increase in mental health score (OR = 1.0, CI: 1.0–1.09, R^2^_Tjur_ = 0.1) and being a health care student (IR = 2.95, CI: 1.53–5.87) were associated with higher likelihood of an upper body injury. On the other hand, an increase in the academic year of study was associated with a 28% decreased likelihood of an upper body MSKD in females (OR = 0.72, CI: 0.58–0.89). There were no significant predictors for upper body MSKDs in males (R^2^_Tjur_ = 0.04).

2. Lower body.

There were no significant predictors of lower body MSKDs in the overall data (R^2^_Tjur_ = 0.02). *When stratified by sex*, there were no significant predictors of lower body injuries in females (R^2^_Tjur_ = 0.02). In males, having regular sports activity was associated with a 536% increase in the likelihood of a lower body injury (OR = 6.36, CI: 2.24–20.76, R^2^_Tjur_ = 0.20).

3. Spine.

There were no significant predictors of spine injury in the overall dataset (R^2^_Tjur_ = 0.01). In females, higher DASS-14 score was associated with a higher likelihood of spine pain (OR = 1.05, CI: 1.01–1.09, R^2^_Tjur_ = 0.03). In males, an increase in hours of sleep was associated with a decrease in likelihood of spine injuries (OR = 0.64, CI: 0.39–0.98, R^2^_Tjur_ = 0.08).

Some predictors were not included in the model due to the non-linear relationship between the logit of the predictor and the outcome.

**Table 7:** Yearly predictors of MSKDs in spine, lower body, and upper body in 2018

	Upper Body			Lower Body			Spine		
	Overall Data (n = 286, R^2^_Tjur_ = 0.09)	Female (n = 20, R^2^_Tjur_ = 0.1)	Male (n = 79, R^2^_Tjur_ = 0.04)	Overall Data n = 89, R^2^_Tjur_ = 0.02	Female (n = 209, R^2^_Tjur_ = 0.02)	Male (n = 78, R^2^_Tjur_ = 0.20)	Overall Data n = 288, R^2^_Tjur_ = 0.01	Female (n = 209, R^2^_Tjur_ = 0.03)	Male (n = 80, R^2^_Tjur_ = 0.08)
**Predictors**	*Incidence Ratio* *(Confidence Interval)*	*Incidence Ratio* *(Confidence Interval)*	*Incidence Ratio* *(Confidence Interval)*	*Incidence Ratio* *(Confidence Interval)*	*Incidence Ratio* *(Confidence Interval)*	*Incidence Ratio* *(Confidence Interval)*	*Incidence Ratio* *(Confidence Interval)*	*Incidence Ratio* *(Confidence Interval)*	*Incidence Ratio* *(Confidence Interval)*
Mental health (DASS-14)	1.05* **(1.01–1.09)**	1.04* **(1.00–1.09)**	-	1.03 (1.00-1.07)	1.03 (1.00–1.08)	-	-	1.05* **(1.01–1.09)**	Not included^†^
Study pressure	-	-	-	-	-	-	-	Not included^†^	-
Health care student (Yes)	2.51* **(1.48–4.35)**	2.95* **(1.53–5.87)**	-	1.53 (0.94–2.50)	-	-	-	4.69 (0.92–3.11)	-
Hours of part-time work per week	-	-	-	-	-	1.08 (1.00–1.18)	-	-	Not included^†^
Last academic year of study (1–5+)	0.77* **(0.64–0.91)**	0.72* **(0.58–0.89)**	-	-	-	-	-	-	-
Last week’s average sleep	-	-	-	-	-	-	-	-	0.64* **(0.39–0.98)**
Hours of cell phone browsing	-	Not included^†^	-	-	-	-	0.92 (0.83–1.01)	Not included^†^	0.81 (0.63–1.02)
Hours of computer use	-	-	-	-	-	-	-	-	-
Regular sport activity (Yes)	1.84* **(1.10–3.11)**	1.79. (0.96–3.39)	2.39. (0.94–6.21)	-	-	6.36* **(2.24–20.76)**	-	-	-

† non-linear relationship with the logit of outcome

* *P < 0.05*

## Discussion

Our study investigated the prevalence and potential risk factors for MSKDs in course-based programs at a Canadian university. The most prevalent sources of self-reported pain over the past 7 days and the past 12 months were at the lower back and neck in all four samples, which is consistent with previous results in university students [4]. In addition, previous studies have found that MSK pain sites are more common in women compared to men [21]; however, we found no significant differences between the two sexes. Our results demonstrated that higher DASS-14 scores were associated with higher 7-day and 12-month prevalence of MSKDs. The subgroup analysis showed that poor mental health was a predictor for higher number of pain sites in the last week and the last 12 month among female students. In addition, we found that regular involvement in sports activity, older age, decreased sleep quantity, lower pressure scores, and being a healthcare student were significantly associated with higher MSKDs.

Interestingly, there was a slight decrease in reported MSKDs in 2020 and 2021, which may be due to the COVID-19 pandemic and potentially the decrease in contact sports (i.e., as per male risk factors). The relationship between COVID and MSKs is a complex and an evolving area of study. Both the COVID-19 infection and its vaccination have been linked to headaches through immune-mediated mechanisms [22], and remote learning has been linked to increased neck pain in university students, which was not observed in this study [23]. More data is needed to understand the role of COVID in MSK pain university students.

Although we conducted yearly cross-section analysis, this multiyear study provides stronger evidence than a single year study. By taking the survey in multiple years, we have a better picture of the most likely risk factors. For example, mental health was not associated with the number of painful sites in male in 2018, but it was related in 2019. Similarly, age was not a factor in 2018, but a factor in 2019. This may be due to the characteristics of our sample and may indicate weak associations. This study provides multiple sampling over several years to draw better conclusions regarding factors associated with MSKDs in university students.

With regards to mental health, it is noteworthy that despite the significant results, an increase in DASS-14 score by one point was associated with only a 2–3% increase in the risk of having MSKDs in the last week or the last year. As such, the difference may not be clinically significant. In males, mental health was a significant predictor of MSKDs in 2019, but not in 2018. The relationship between sex, mental health, and its correlation with MSK pain has shown conflicting evidence in the literature. Some studies have suggested that women are more likely to report depression and higher pain severity of chronic pain than men [15]; however, Hu et al. (2021) found that males had a stronger correlation between knee pain and depression as compared to females [24]. Considering the low response rate from male students, further studies with more representation are warranted to understand the role of mental health in MSKDs and how this relationship differ between sexes.

Another predictor of interest was regular sports activity. While a previous small-scale study found that regular sports activity is associated with a decrease in MSKDs [4], a large-scale epidemiological study involving over 3,000 university students revealed that longer sports participation hours (including combat sports, yoga, basketball, and soccer) were associated with more knee symptoms [11]. Our results corroborated that having a regular sports activity increased the risk of lower body injuries in males by 536%. Overall, physical activity increased the prevalence of MSKDs more in the lower body than the upper body. This can be attributed to the fact that organized sports activity at the university level are of moderate and high intensity, which is linked to increased incidence of acute MSKDs [25–27]. More than two thirds of injuries in adolescent athletes occur in the lower body, notably the ankles and the knees [27], which could explain the high rate of lower body injuries in students with sports activity in our study. However, it remains unclear how sports involvement at the university level can influence the risk of chronic MSKDs.

Previous research has suggested that older age is associated with a higher prevalence of MSKDs [28]. Given that the age of our respondents ranged from 18 to 50 years (median 23; IQR 5), it is probable that the differences may be attributed to age-related physiologic changes. Additionally, senior students may experience more anxiety or distress related to the increased responsibilities in student bodies or job hunting, which may also contribute to their higher prevalence of MSKDs.

Peer, academic, and family pressure constituted to the pressure score, which was a significant predictor of less MSK pain in male university students. Previous studies revealed that there was a significant positive correlation between academic stress and MSKDs in college students, and the prevalence of MSKDs increased during exam seasons [29, 30]. However, we found that increased pressure scores were associated with 4–5% decrease in yearly and weekly pain sites in males in 2019. Our finding suggested that an increase in each pressure score only resulted in a small decrease in MSKDs. As such, this relationship may not have significant real-world implications. More data is needed to further understand how pressure affects MSKDs in university students or assess whether the association is U-shaped.

Less hours of sleep have been reported to be linked to more MSK pain in the neck, lower back, and knees in high school students [31]. Our study found that an additional hour of sleep was associated with 36% less likelihood of spine pain in males. Previous research in children and adolescents have suggested that poor sleep quality and quantity were strongly associated with neck pain onset [32]. Insomnia has been found to be an independent predictor of chronic low back pain. The negative effects of sleep disturbance on pain may be ascribed to the increased central/peripheral pain sensitivity [33, 34]. That said, it is noteworthy that quantity and quality of sleep can be influenced by other risk factors. Students with chronic insomnia is known to have poorer mental health, including increased anxiety, depression, and stress [35]. As such, while the effects of sleep on neck pain are consistent with previous research, in the context of university students, it is important to consider that mental health and academic stressors may affect sleep duration and quality.

We hypothesized that being a health care student would be associated with a higher prevalence of MSKDs as compared to non-healthcare students based on previous research [5]. We also found that healthcare students had a higher likelihood of upper body pain, especially in females, where it increased the likelihood of MSKDs by 195%. It has been suggested that longer studying time, poor posture while studying, high levels of stress, or clinical placement may be contributing to an increase in MSK pain in healthcare students [4, 9].

This is the first study investigating the prevalence and risk factors for musculoskeletal injuries and pain sites among university students in Canada. The high prevalence of MSKDs in all four years of samples highlights that MSKDs frequently occur during university years. Considering that MSKDs in younger ages can increase the risk of MSKDs in later life [3], recognizing these risk factors is an important first-step in reducing the impact and occurrence of MSKDs in university students.

### Limitations

This survey was initially designed to be a longitudinal survey, however, due to reliance on participant-provided email addresses, we were unable to accurately match participants’ responses across years. As such, the responses represent only a subset of McMaster students across several years. Future large-scale studies should involve more universities across Canada to identify potential risk factors for pain sites in university students.

Since we initially were not aware of potential different risk factors for male and female respondents, an overall analysis was planned. Future studies should involve a larger number of male students to better understand their risk factors and the potential differences between sexes. Future research should also explore how and why the risk factors differ between the sex. Further risk factors, including co-morbidities such as congenital or autoimmune conditions, can also be explored in future studies. A longitudinal study is warranted to better understand differences in prevalence and risk factors between chronic and acute MSKDs.

## Conclusions

Our study has suggested that neck and lower back pain are highly prevalent in university students and are the most common sites of pain. Poorer mental health, increased pressure, lack of sleep, regular involvement in sports activity, and being a healthcare are associated with increased prevalence of MSKDs in university students. Future studies should investigate how public health measures such as increased access to mental health services and increased awareness may impact MSK pain. Additionally, more attention should be given to addressing program-specific risk factors, especially in healthcare students, to effectively reduce their prevalence of MSKDs.

## Electronic supplementary material

Below is the link to the electronic supplementary material.

Supplementary Material 1

## Data Availability

All data used and/or analyzed during the current study are available from the corresponding author on reasonable request.

## Abbreviations

AIC: Akaike information criterion
BIC: Bayesian information criterion
CI: confidence interval
DASS-14: Depression, Anxiety, and Stress Scale-14
IQR: interquartile range
MSK: Musculoskeletal
MSKDs: Musculoskeletal disorders
