# Supplementary material for: Prevalence of musculoskeletal disorders and associated risk factors in canadian university students
Source: BMC Musculoskelet Disord. 2023 Jun 19;24:501. doi: 10.1186/s12891-023-06630-4 (PMC10278339; doi:10.1186/s12891-023-06630-4)
Supplement: Supplementary file 1 — Supplementary Material 1 [file 12891_2023_6630_MOESM1_ESM.docx]

## Table 1A - Characteristics of sex-stratified data

|  | **Female** | | | | **Male** | | | |
| --- | --- | --- | --- | --- | --- | --- | --- | --- |
| **Sociodemographic data** | **Year 2018**  **(n = 209)** | **Year 2019**  **(n = 120)** | **Year 2020**  **(n = 88)** | **Year 2021**  **(n = 54)** | **Year 2018**  **(n = 80)** | **Year 2019**  **(n = 47)** | **Year 2020**  **(n = 42)** | **Year 2021**  **(n = 20)** |
| Mean age (in years) | 22.2 ± 4.2 | 23.3 ± 4.4 | 24.1 + 3.8 | 24.9 ± 3.9 | 20.8 ± 2.1 | 21.9 ± 2.2 | 23.0 ± 2.3 | 24.1 ± 2.5 |
| Mean job hours | 5.7 ± 10.2 | 2.4 ± 5.5 | 3.5 ± 5.6 | 5.8 ± 9.8 | 3.9 ± 6.9 | 3.8 ± 6.6 | 2.3 ± 5.1 | 0.6 ± 1.8 |
| Year of study (0, 1, 2, 3, 4) | | | | | | | | |
| 0 | 47 (26.5%) | 3 (2.5%) | 1 (1.1%) | 2 (3.7% | 17 (21.3%) | 3 (6.4%) | 0 | 0 |
| 1 | 55 (31%) | 34 (28.3%) | 2 (2.2%) | 3 (5.5%) | 15 (18.8%) | 13 (27.7%) | 4 (9.5%) | 2 (10%) |
| 2 | 33 (18.6%) | 39 (32.5%) | 30 (34%) | 2 (3.7%) | 14 (17.5%) | 10 (21.3%) | 13 (30.9%) | 1 (10%) |
| 3 | 40 (22.6%) | 26 (21.7%) | 26 (29.5%) | 13 (24.1%) | 21 (26.5%) | 9 (19.1%) | 10 (23.8%) | 4 (20%) |
| 4 | 24 (13.6%) | 8 (6.7%) | 18 (20.4%) | 16 (29.6%) | 8 (10%) | 9 (19.1%) | 6 (14.3%) | 5 (25%) |
| >5 | 8 (4.5%) | 9 (7.5%) | 11 (12.5%) | 14 (26%) | 8 (6.25%) | 2 (4.3%) | 8 (19%) | 7 (35%) |
| Type of study program | | | | | | | | |
| Healthcare | 114 | 68 | 50 | 30 | 26 | 21 | 21 | 12 |
| Non-healthcare | 95 | 51 | 38 | 24 | 54 | 26 | 21 | 8 |
| Habits | | | | | | | | |
| Smoking (No) | 193 (92.3%) | 113 (94.2%) | 84 (95%) | 52 (96.2%) | 73 (91.3%) | 43 (91.5%) | 37 (88.1%) | 20 (100%) |
| Regular exercise (Yes) | 104 (49.7%) | 55 (45.8%) | 36 (40.9%) | 16 (29.6%) | 50 (62.5%) | 29 (61.7%) | 18 (42.8%) | 13 (65%) |
| Average hours of sleep over the last week | 6.8 ± 1.7 | 6.6 ± 1.2 | 6.98 ± 1.2 | 6.7 ± 1.0 | 6.7 ± 1.13 | 6.7 ± 1.1 | 7.2 ± 1.9 | 6.7 ± 1.0 |
| Hours of computer usage per day | 6 ± 3.2 | 5.6 ± 2.7 | 6.1 ± 3.2 | 6.2 ± 3.0 | 5.3 ± 3.1 | 5.3 ± 2.9 | 5.8 ± 2.7 | 4.4 ± 2.2 |
| Hours of cellphone usage per day | 3.2 ± 2.7 | 3.2 ± 2.3 | 3.6 ± 1.9 | 3.4 ± 1.9 | 3.2 ± 2.1 | 3.2 ± 1.9 | 4.4 ± 3.8 | 3.4 ± 2.0 |
| Psychological factors | | | | | | | | |
| Depression, Anxiety Stress Scale DASS-14 (14-30) | 25.1 ± 8.0 | 22.6 ± 6.9 | 22.9 ± 6.9 | 21.0 ± 6.1 | 14.4 ± 6 | 14 ± 6.6 | 15.1 ± 5.6 | 11.2 ± 5.4 |
| Total pressure score from family, peer, and studies (1-30) | 15.6 ± 6 | 14.8 ± 6.1 | 14.7 ± 6.1 | 14.3 ± 6.1 | 22.6 ± 6.7 | 20.6 ± 6.5 | 22.2 ± 8.4 | 20 ± 7.0 |

## Table 2A. Prevalence of pain sites by body part

|  | Head  (n, %) | Neck  (n, %) | Upper  Back  (n, %) | Lower  Back  (n, %) | Shoulder  (n, %) | Elbow  (n, %) | Wrist/  Hands  (n, %) | Pelvis/  Groin  (n, %) | Hips/  Thighs  (n, %) | Knees  (n, %) | Lower  Legs  (n, %) | ankles/feet/toes  (n, %) |
| --- | --- | --- | --- | --- | --- | --- | --- | --- | --- | --- | --- | --- |
| **Year 2018** | | | | | | | | | | | | |
| Currently | 13 (4.4) | 54 (18.2) | 60 (20.2) | 68 (22.9) | 54 (18.2) | 5 (1.68) | 25 (8.42) | 7 (2.34) | 29 (9.76) | 26 (8.75) | 21 (7.07) | 42 (14.1) |
| Past 7 days | 42 (14.1) | 89 (30.0) | 79 (26.6) | 101 (34.0) | 84 (28.3) | 7 (2.34) | 48 (16.2) | 11 (3.70) | 46 (15.5) | 56 (18.9) | 33 (11.1) | 57 (19.2) |
| Past 12 months | 71 (23.9) | 134 (45.1) | 112 (37.7) | 164 (55.2) | 124 (41.8) | 24 (8.08) | 96 (32.3) | 21 (7.07) | 74 (25.0) | 94 (31.6) | 54 (18.2) | 93 (31.3) |
| Most significant | 22 (7.4) | 50 (16.8) | 49 (16.5) | 100 (33.7) | 48 (16.2) | 4 (1.34) | 30 (10.1) | 8 (2.67) | 25 (8.42) | 36 (12.1) | 17 (5.72) | 45 (15.2) |
| **Year 2019** | | | | | | | | | | | | |
| Currently | 10 (5.8) | 27 (15.6) | 26 (15.0) | 28 (16.2) | 24 (13.9) | 2 (1.16) | 18 (10.4) | 4 (2.31) | 8 (4.62) | 15 (8.67) | 4 (2.3) | 11 (6.4) |
| Past 7 days | 22 (12.7) | 41 (23.7) | 32 (18.5) | 40 (23.1) | 34 (19.7) | 5 (2.89) | 28 (16.2) | 5 (2.89) | 18 (10.4) | 22 (12.7) | 11 (6.4) | 14 (8.1) |
| Past 12 months | 33 (19.1) | 56 (32.4) | 50 (28.9) | 70 (40.5) | 54 (31.2) | 12 (6.93) | 48 (27.7) | 13 (7.51) | 28 (16.2) | 47 (27.2) | 17 (9.8) | 32 (18.5) |
| Most significant | 12 (6.9) | 29 (16.8) | 22 (12.7) | 37 (21.4) | 28 (16.2) | 0 (0.0) | 22 (12.7) | 5 (2.89) | 10 (5.78) | 13 (7.51) | 8 (4.6) | 14 (8.1) |
| Year 2020 | | | | | | | | | | | | |
| Currently | 12  (9.2) | 29  (22.1) | 21  (16.0) | 35  (26.7) | 21  (16.0) | 2  (1.5) | 14  (10.7) | 2  (1.5) | 7  (5.3) | 4  (3.1) | 2  (1.5) | 5  (3.8) |
| Past 7 days | 19  (14.5) | 38  (29.0) | 31  (23.7) | 38  (29.0) | 30  (22.9) | 5  (3.8) | 21  (16.0) | 4  (3.1) | 18  (13.7) | 15  (11.5) | 4  (3.1) | 13  (9.9) |
| Past 12 months | 33  (25.2) | 55  (42.0) | 54  (41.2) | 64  (48.9) | 52  (39.7) | 11  (8.4) | 38  (29.0) | 8  (6.1) | 29  (22.1) | 32  (24.4) | 12  (9.2) | 26  (19.8) |
| Most significant | 12  (9.2) | 24  (18.3) | 22  (16.8) | 35  (26.7) | 24  (18.3) | 2  (1.5) | 16  (12.2) | 2  (1.5) | 11  (8.4) | 9  (6.9) | 3  (2.3) | 9  (6.9) |
| Year 2021 | | | | | | | | | | | | |
| Currently | 6  (7.9) | 13  (17.1) | 18  (23.7) | 17  (22.4) | 9  (11.8) | 2  (2.6) | 4  (5.3) | 0  (0.0) | 5  (6.6) | 3  (3.9) | 0  (0.0) | 6  (7.9) |
| Past 7 days | 10  (13.2) | 22  (28.9) | 22  (28.9) | 24  (31.6) | 14  (18.4) | 3  (3.9) | 7  (9.2) | 2  (2.6) | 14  (18.4) | 9  (11.8) | 3  (3.9) | 13  (17.1) |
| Past 12 months | 19  (25.0) | 29  (38.2) | 31  (40.8) | 39  (51.3) | 28  (36.8) | 6  (7.9) | 27  (35.5) | 4  (5.3) | 22  (28.9) | 15  (19.7) | 6  (7.9) | 19  (25.0) |
| Most significant | 4  (5.3) | 11  (14.5) | 17  (22.4) | 20  (26.3) | 13  (17.1) | 1  (1.3) | 7  (9.2) | 1  (1.3) | 9  (11.8) | 5  (6.6) | 3  (3.9) | 8  (10.5) |

## Table 3A. Summary of overall pain sites in females and males

|  | Pain Sites in Females | | | | Pain Sites in Males | | | |
| --- | --- | --- | --- | --- | --- | --- | --- | --- |
| Data | Year 2018  (n = 209) | Year 2019  (n = 120) | Year 2020  (n = 88) | Year 2021  (n = 54) | Year 2018  (n = 80) | Year 2019  (n = 47) | Year 2020  (n = 42) | Year 2021  (n = 20) |
|  | Mean  (SD) | Mean  (SD) | Mean  (SD) | Mean  (SD) | Mean  (SD) | Mean  (SD) | Mean  (SD) | Mean  (SD) |
| Number of pain sites (yearly) | 3.5 ± 2.4 | 3.3 ± 2.6 | 3.2 ± 2.5 | 3.2 ± 2.2 | 3.0 ± 2.5 | 2.7 ± 2.2 | 2.2 ± 1.9 | 2.4 ± 1.4 |
| Number of pain sites (weekly) | 2.1 ± 1.8 | 2.0 ± 1.9 | 1.8 ± 1.8 | 1.9 ± 1.7 | 1.8 ± 1.7 | 1.6 ± 1.5 | 1.3 ± 1.4 | 1.4 ± 1.1 |
| Injury by body group | | | | | | | | |
| Spine (Yes) n(%) | 141 (67.5%) | 76 (63.3%) | 56 (63.6%) | 37 (68.5%) | 52 (65%) | 28 (59.6%) | 26 (61.9%) | 15 (75%) |
| Lower Body (Yes) n(%) | 137 (65.6%) | 68 (56.7%) | 46 (52.3%) | 27 (50%) | 41 (51.3%) | 22 (46.8%) | 14 (33.3%) | 11 (55%) |
| Upper Body (Yes) n(%) | 144 (68.9%) | 78 (65%) | 57 (64.8%) | 39 (72.2%) | 47 (58.8%) | 30 (63.8%) | 15 (35.7%) | 10 (50%) |
